# The Expression of a Germline Fusion Gene Involving a Protein-Coding and a Long Non-Coding RNA Gene Results in Severe Brain Malformations

**DOI:** 10.3390/genes16050598

**Published:** 2025-05-18

**Authors:** Lukas Kaufmann, Christine Beichler, Jasmin Blatterer, Ingrid Janisch, Bence Csapó, Elisabeth Schreiner, Sarah Verheyen, Jochen B. Geigl, Christian Windpassinger

**Affiliations:** 1 Diagnostic and Research Institute of Human Genetics, Medical University of Graz, 8010 Graz, Austria; christine.beichler@medunigraz.at (C.B.); jasmin.blatterer@medunigraz.at (J.B.); ingrid.janisch@medunigraz.at (I.J.); elisabeth.schreiner@medunigraz.at (E.S.); sarah.verheyen@medunigraz.at (S.V.); christian.windpassinger@medunigraz.at (C.W.); 2 Division of Obstetrics, Department of Obstetrics and Gynaecology, Medical University of Graz, 8010 Graz, Austria; b.csapo@frauenschall.at

**Keywords:** gene fusion, MN1, lncRNA, brain malformations

## Abstract

In the present study, an exceptional germline gene fusion involving the protein-coding *MN1* gene and the long non-coding RNA (lncRNA) gene *CPMER* was detected as the genetic cause of severe cerebral abnormalities with unfavorable prognosis in a male fetus at 14 weeks of gestation. Quantitative and qualitative RNA analyses indicate the expression of C-terminally truncated MN1 proteins. MN1 proteins lacking the C-terminal amino acids have been previously described to cause an ultra-rare syndrome with brain malformations due to a gain-of-function effect. To the best of our knowledge, this is the first study reporting a germline gene fusion of a protein-coding gene and an lncRNA gene linked to a functional, but neomorphic, protein associated with severe phenotypic abnormalities. The results of our study are not only relevant for the genotype–phenotype correlation of *MN1* but should especially raise awareness for potentially disease-associated protein expressions in germline gene fusions involving lncRNAs.

## 1. Introduction

Gene fusions can be the result of chromosomal rearrangements such as deletions, translocations, tandem duplications, or inversions, as well as the splicing of a chimeric pre-mRNA [1]. Fusions involving long non-coding RNA (lncRNA) genes are not yet well understood but are thought to be highly relevant in tumorigenesis. Recently, it has been discovered that fusions of protein-coding genes (PCGs) with lncRNA genes can even be translated into functional proteins. However, this phenomenon has only been described in somatic cancer cells so far [2,3].

MN1 C-terminal truncation (MCTT) syndrome is a very rare disease with fewer than 30 cases described worldwide, characterized by craniofacial defects, expressive language delay, impaired intellectual development, dysmorphic ears, and distinct structural brain abnormalities [4]. The syndrome is caused by truncating variants in *MN1* through a gain-of-function effect, mediated by the expression of MN1 proteins lacking the C-terminal amino acids encoded by exon 2 [5,6]. The *MN1 proto-oncogene, transcriptional regulator* (*MN1*) is a protein-coding gene located in the chromosomal region 22q12.1. *MN1* comprises only two exons, with the majority (95%) of the 1320 amino acid long MN1 protein being encoded by exon 1 [7]. To date, exclusively C-terminal nonsense and frameshift variants escaping nonsense-mediated mRNA decay have been reported in association with MCTT syndrome [5,6].

In the present study, an exceptional germline gene fusion involving *MN1* and an lncRNA was detected in a male fetus displaying severe cerebral abnormalities.

## 2. Materials and Methods

### 2.1. Ethics Statement

This study was approved by the ethics committee of the Medical University of Graz, Austria (approval number: 35-476 ex 22/23). Written informed consent was obtained from the mother of the fetus.

### 2.2. Clinical Evaluation and Diagnosis

The initial referral of the pregnant woman and the findings of the sonographic examination of the fetus were provided by experts of the Department of Obstetrics and Gynaecology at the University Hospital of Graz, Austria.

### 2.3. DNA and RNA Isolation

DNA isolation was carried out with phenol–chloroform from chorionic villus using a standard protocol. RNA was isolated from chorionic villus culture using TRIzol reagent (Invitrogen™/Thermo Fisher Scientific, Waltham, MA, USA). Some of the cultures were pre-treated with puromycin solution (catalog no. P8833, Sigma-Aldrich, Burlington, MA, USA) to inhibit nonsense-mediated mRNA decay (NMD), according to the standard operating procedure (SOP) “RNA Analyse nach KurzzeitKul_Trizol_isol” of our institute.

### 2.4. SNP Array

Copy number variation analysis was performed using the Infinium CytoSNP-850K (Illumina, San Diego, CA, USA) in combination with the BlueFuse™ Multi Analysis Software v4.5 (Illumina). The obtained data were also analyzed using the UCSC Genome Browser (www.genome.ucsc.edu, accessed on 24 November 2024) [8] and the DECIPHER database [9,10].

### 2.5. Quantitative Real-Time PCR

Quantitative real-time PCR (qRT-PCR) analysis from DNA was performed using a 7500 Fast Real-Time PCR System (Applied Biosystems™/Thermo Fisher Scientific, Waltham, MA, USA). With qRT-PCR from DNA, confined placental mosaicism was excluded, the size of the deletion detected by SNP array was confirmed, and segregation analysis on the parents of the fetus was performed. The region-specific primer sets used are listed in Supplementary Table S1.

For quantitative RNA analysis, qRT-PCR from cDNA was performed. RNA was quantified using the Qubit™ RNA Broad Range Assay Kit (catalog no. Q10210, Invitrogen™/Thermo Fisher Scientific): RNA_NMD not inhibited = 145 ng/μL and RNA_NMD inhibited = 179 ng/μL. The integrity of the RNA was then checked using the Bioanalyzer RNA 6000 pico assay (catalog no. 5067-1513, Agilent, Santa Clara, CA, USA) according to the manufacturer’s protocol, and the 18S and 28S RNA peaks were visible. Subsequently, cDNA synthesis was conducted by reverse transcription with the Omniscript RT Kit (catalog no. 205113, Qiagen, Hilden, Germany) according to the manufacturer’s protocol, using 870 ng of RNA_NMD not inhibited and 895 ng of RNA_NMD inhibited. The qRT-PCR analysis of the cDNAs of RNA_NMD not inhibited, RNA_NMD inhibited, and the Human Reference RNA (catalog no. 750500, Agilent) was performed with the StepOnePlus™ Real-Time PCR System (catalog no. 4376600, Applied Biosystems™/Thermo Fisher Scientific), using a standard protocol with 40 cycles, as well as melting curve analysis. The amplicon of exon 1 of *MN1* was used as a comparative control for the quantitation of comparative Ct (delta–delta Ct values) analysis. The primer sets used for quantitative RNA (cDNA) analysis are listed in Supplementary Table S2.

### 2.6. Sanger Sequencing of cDNA

QuantiTect Reverse Transcription Kit (catalog no. 205311, Qiagen) was used for cDNA synthesis according to the manufacturer’s protocol. Subsequent amplification of the cDNA was performed using AmpliTaq GoldTM 360 Master Mix (catalog no. 4398881, Applied Biosystems™/Thermo Fisher Scientific) according to the SOP “AmpliTaq Gold 360 MasterMix PCR” of our institute. The primer sets listed in Supplementary Table S3 were used for Sanger sequencing of the cDNA samples “RNA_NMD not inhibited” (without prior puromycin treatment), “RNA_NMD inhibited” (with prior puromycin treatment), and the Human Reference RNA (catalog no. 750500, Agilent), which was utilized as control sample. The primer sets “sp_MN1_E1_v2_f” and “sp_MN1_E1_r” were used as a positive control, as exon 1 of the *MN1* gene is not affected by the deletion. The wild-type sequence of this region in exon 1 was confirmed in both RNA_NMD not inhibited and RNA_NMD inhibited, as well as in the control sample. The remaining primer sets were used to detect possible fusion genes and their expression. In addition, information on splicing was obtained. For protein sequence alignment, the analysis tool Clustal Omega v2.1 [11] was used.

### 2.7. Trio-Whole-Exome Sequencing

Trio-whole-exome sequencing (Trio-WES) was performed in addition to exclude the possibility of other pathogenic variants in protein-coding genes that could be the cause of the fetal phenotype abnormalities. Trio-WES involves the sequencing of all protein-coding regions of the genome as well as the mitochondrial DNA, with subsequent phenotype-based screening and comparison with the parents’ data. Variant prioritization was based on the HPO terms: intrauterine growth retardation (HP:0001511), decreased thalamic volume (HP:0012695), encephalocele (HP:0002084), intracranial cystic lesion (HP:0010576), aplasia/hypoplasia of the brainstem (HP:0007362) and ventriculomegaly (HP:0002119). The specific regions were enriched using Twist Human Core Exome and Twist Mitochondrial Panel (TWIST Bioscience, South San Francisco, CA, USA). Sequencing was performed on the NextSeq 550 Sequencing System (Illumina, followed by variant annotation and analysis using VarSeq (Golden Helix, Bozeman, MT, USA).

## 3. Case Report

### 3.1. Clinical Description

We investigated the genetic cause of cerebral malformations with unfavorable prognosis in a male fetus at 14 weeks of gestation.

**Figure 1:**
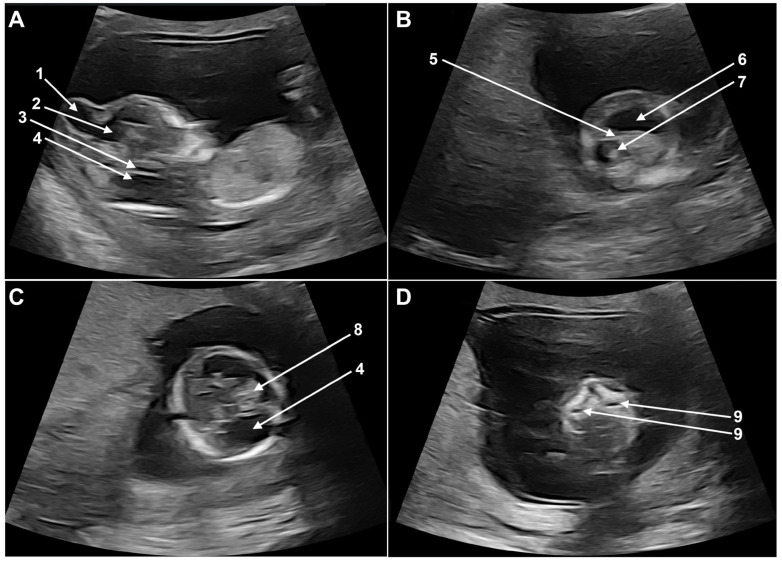
Fetal ultrasound images at the time of the first-trimester scan: (**A**) 1 + 2: cranial encephalocele and communication to right ventricle; 3: hypoplastic brain stem; 4: enlarged posterior fossa. (**B**) 5: asymmetric cerebral midline; 6: dilated left lateral ventricle; 7: right ventricle. (**C**) 8: choroid plexus; 4: enlarged posterior fossa. (**D**) 9: prominent maxillary cavities.

Fetal ultrasound examination at the time of first-trimester scan revealed a severely disrupted cerebral and cerebellar development. The most striking anomaly was the presence of a large cranial encephalocele communicating with the right lateral ventricle (Figure 1A). The cerebral symmetry was disrupted, the left lateral ventricle was dilated, the thalamic area was hypoplastic, and the choroid plexus was small and shifted cranially and anteriorly (Figure 1B,C). The posterior fossa was missing the normal landmarks: the brain stem was hypoplastic, the fourth ventricle (intracranial translucency) could not be identified, the brain stem skull distance was severely enlarged, and the structures of the posterior fossa were replaced by a sonolucent area (Figure 1A,C). The maxillary cavities were prominent, unusual for this gestational age, and bilateral cleft palate could not be excluded (Figure 1D). No further anomalies of the fetus were identified.

The pregnancy was terminated in the 15th week of gestation due to the severity of the ultrasound findings.

### 3.2. DNA Analyses

**Figure 2:**
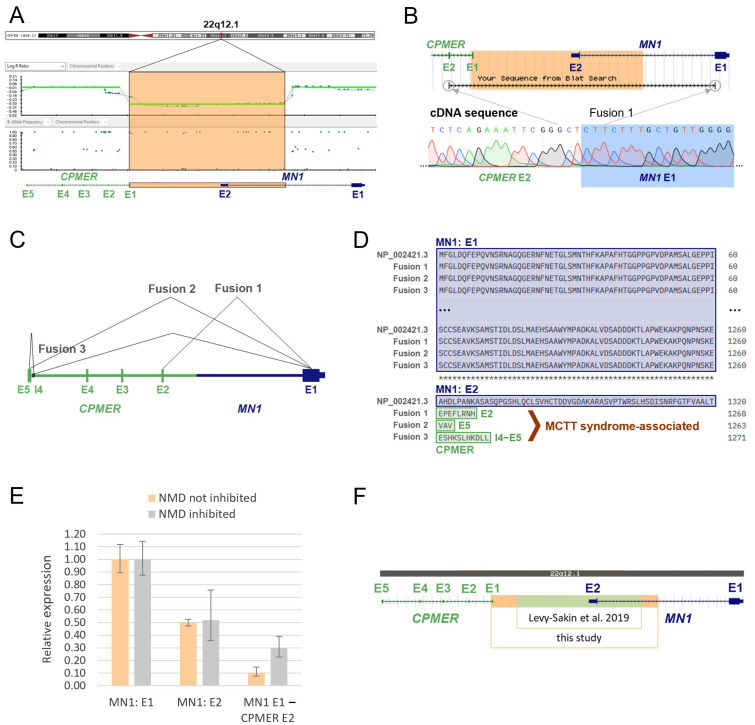
Analysis of a germline gene fusion in a fetus with severe brain malformations, involving a protein-coding (*MN1*) gene and a long non-coding RNA (*CPMER*) gene, indicates expression of disease-causing truncated proteins: (**A**) The detection of a heterozygous de novo deletion with breakpoints in the respective intron 1 of the genes *MN1* and *CPMER* in the fetal germline. The deleted region chr22:27,714,323-27,772,264 (GRCh38/hg38) is highlighted in orange. (**B**) Sanger sequencing of cDNA reveals a direct linkage between the first exon of *MN1* and exon 2 of *CPMER*. (**C**) Illustration of the identified RNA fusion transcripts and the related alternative splicing of the fusion pre-mRNA: Fusion 1: *MN1* E1-*CPMER* E2, Fusion 2: *MN1* E1-*CPMER* E5, and Fusion 3: *MN1* E1-*CPMER* I4 (short segment)-*CPMER* E5. The respective junctions could be detected under both conditions, the inhibition of nonsense-mediated mRNA decay (NMD) as well as without NMD inhibition. No fusion transcripts were detected in controls. (**D**) In silico protein sequence alignment indicates the C-terminally truncated MN1 proteins associated with the identified fusion transcripts. The sequence of the MN1 wild-type protein NP_002421.3 is depicted as well, where the last 60 of the 1320 amino acids are encoded by exon 2. The expression of MN1 proteins lacking the amino acids encoded by exon 2 is causative for the *MN1* C-terminal truncation (MCTT) syndrome via the gain-of-function effect [5]. (**E**) Quantitative RNA analysis indicates an almost equal expression of wild-type and *MN1-CPMER* fusion allele in the fetus. The analysis was performed under two conditions: NMD inhibition and without NMD inhibition. The expression levels of *MN1* exon 1 (MN1: E1) were defined as reference values, as they are not specific for a certain allele. Expression of the wild-type-specific exon 2 amplicon (MN1: E2) was about half as high as exon 1 expression under standard conditions (NMD not inhibited). Accordingly, the wild-type allele and the gene fusion allele were expressed at almost the same level in the fetus. An expression level of less than 50% was observed for the fusion transcript *MN1* E1-*CPMER* E2 under both conditions, reflecting the presence of multiple fusion transcripts. (**F**) Levy-Sakin et al. detected a different heterozygous exon 2 deletion of *MN1* in an apparently healthy individual from the 1000 Genomes Project’s sample pool [12]. Compared to the fetal deletion (highlighted in orange), the deleted region chr22:27,723,174-27,766,326 (GRCh38/hg38) in the unaffected individual (highlighted in green) does not lead to the formation of a gene fusion with *CPMER*.

SNP array analysis after chorionic villus sampling revealed a heterozygous de novo deletion in the chromosomal region 22q12.1 with a size of 58 kb (arr[GRCh38] 22q12.1(27710404x2, 27714323_27772264x1, 27772359x2)). The possibility of confined placental mosaicism was ruled out by also detecting the deletion in the DNA derived from fetal tissue after medically indicated termination of pregnancy. The deletion breakpoints were each located in intron 1 of the affected genes *CPMER* (lncRNA) and *MN1* (protein-coding), indicating a possible gene fusion on the negative DNA strand (Figure 2A).

Additional trio-whole-exome sequencing and chromosome analysis did not reveal any evidence of another potentially causative variant in the fetus.

### 3.3. RNA/cDNA Analyses

To verify whether the *MN1*-*CPMER* fusion gene is actually transcribed into RNA, cDNA analyses were performed. Sanger sequencing of the cDNA revealed a direct link between exon 1 of *MN1* and exon 2 of *CPMER* (Figure 2B). In addition, two further direct junctions between the first *MN1* exon and *CPMER* were detected in the cDNA: one sequence contained a short segment of intron 4, which merged into exon 5, and one sequence showed a direct junction to exon 5 of *CPMER*. The results of these qualitative RNA analyses suggest the alternative splicing of the fusion pre-mRNA (Figure 2C). Each of the RNA transcripts could be detected under both conditions, the inhibition of nonsense-mediated mRNA decay (NMD) and without NMD inhibition. No fusion transcripts were detected in controls.

In silico protein sequence alignment demonstrated that the translation of the three identified fusion transcripts led to shortened *MN1* proteins lacking all amino acids encoded by exon 2 (Figure 2D). Such C-terminally truncated MN1 proteins have been reported to be the cause of the extremely rare MCTT syndrome [5,6].

In addition, quantitative cDNA analyses were performed to quantify the expression level of the MN1-CPMER fusion gene. The amount of *MN1* exon 1 transcribed was set as a reference value, since this exon was expressed in both alleles (wild-type and gene fusion). The expression level of *MN1* exon 2 was about half the level of exon 1 expression under standard conditions (NMD not inhibited), indicating an almost equal expression of wild-type allele and gene fusion allele (Figure 2E). The analysis under NMD inhibition does not allow any conclusions to be drawn in this respect, due to the relatively high standard deviation for exon 2. In addition, the expression of one fusion transcript (*MN1* E1-*CPMER* E2) was also determined quantitatively. An expression level of less than 50% was observed under both conditions, which is consistent with the previous detection of several fusion transcripts and possible alternative splicing (Figure 2E). The remaining two fusion transcripts could not be quantitatively investigated due to the limited amount of primary sample.

### 3.4. Database and Literature Review

A review of the international databases and the literature revealed no evidence for the presence of a comparable deletion with breakpoints in intron 1 of *MN1* and *CPMER*. However, a similar deletion in 22q12.1, which also removed exon 2 of *MN1* but did not result in the formation of a fusion gene, was identified in the germline of an apparently healthy individual from the 1000 Genomes Project (Figure 2F) [12].

## 4. Discussion

In the present study, a 58 kb heterozygous de novo deletion in 22q12.1 affecting the protein-coding gene *MN1* and the lncRNA *CPMER* was detected in a male fetus as the cause of severe cerebral malformations. Our findings demonstrate that the germline deletion results in an *MN1*-*CPMER* gene fusion and the expression of stable mRNAs (Figure 2A–C). Furthermore, we were able to show that the fusion allele is expressed at a similar level as the wild-type allele (Figure 2E). The results also reveal alternative splicing and the expression of several fusion transcripts.

The translation of these mRNAs is expected to result in truncated MN1 proteins lacking the last 60 C-terminal amino acids encoded by exon 2 (Figure 2D). Mak et al. and Miyake et al. have shown in their studies that these C-terminally truncated proteins cause MCTT syndrome via a gain-of-function effect [5,6]. Besides intellectual and motor developmental abnormalities, craniofacial features and distinctive findings on brain imaging are key characteristics of MCTT syndrome [4]. The findings observed in the fetus are in line with the brain abnormalities described in MCTT syndrome, although due to the early stage of fetal development and the fact that the cerebellum is not yet developed, definite conclusions cannot be drawn.

The results of the literature and database review also point to the expression of the *MN1*-*CPMER* fusion gene as the cause of the fetal brain malformations. The deletion reported in the study of Levy-Sakin et al., in an apparently healthy person, without serious phenotypic abnormalities, also affects the second exon of *MN1*. In contrast to the fetal deletion, the deletion detected in this person does not involve the *CPMER* gene, and thus no fusion gene is formed (Figure 2F) [12]. Instead, the deletion is more likely to result in a loss of *MN1* function due to nonsense-mediated mRNA decay. Heterozygous loss-of-function mutations in *MN1* are not associated with facial and brain abnormalities [4]. This provides further evidence that the deletion detected in the fetus does not result in a loss of function but rather in a gain-of-function effect mediated by the expression of C-terminally truncated MN1 proteins.

In addition, it has not yet been reported that mutations in *CPMER*, which encodes the lncRNA Cytoplasmic Mesoderm Regulator, could be associated with brain abnormalities. In their recent study, Lyu et al. found that *CPMER* promotes cardiomyocyte differentiation in mice and humans [13].

In somatic gene fusions of PCGs and lncRNAs, Guo et al. found that the fusion transcripts are subject to regulation by the promoter of the upstream PCG [3]. Accordingly, the promoter of the *MN1* gene is associated with the regulation of the studied *MN1*-*CPMER* gene fusion. *MN1* is expressed in many different tissues, with high expression also in the brain, especially in the cerebral cortex and basal ganglia [14,15]. Unfortunately, we were unable to perform protein expression analysis as fetal tissue samples were not available.

In summary, all the obtained results indicate that the de novo deletion in 22q12.1 and the subsequent expression of the *MN1*-*CPMER* fusion transcript are causative for the fetal brain malformations. To the best of our knowledge, this is the first study reporting a germline gene fusion of a PCG and an lncRNA gene that is associated with a functional protein and related severe phenotypic abnormalities.

As Sánchez-Marín et al. recently stated in their review article, research on gene fusions involving lncRNAs “is still in its infancy”, with current focus solely on somatic cancer cells [2]. The results of our study not only expand the mutation spectrum at the *MN1* locus by deletions resulting in the expression of C-terminally truncated proteins but also have locus-independent relevance. With this study, we want to raise awareness in the genetic community of the possibility that lncRNA fusions may result in protein expression with high clinical significance in the germline as well. Studying protein-forming gene fusions involving genes that are not originally coding genes unlocks novel opportunities in the search for disease-causing alterations.

## Data Availability

The original contributions presented in this study are included in the article/Supplementary Materials. Further inquiries can be directed to the corresponding author.

## Supplementary Materials

The following supporting information can be downloaded at: https://www.mdpi.com/article/10.3390/genes16050598/s1, Table S1. Primer sets for qRT-PCR from DNA. Table S2. Primer sets for qRT-PCR in quantitative RNA analysis. Table S3. Primer sets for Sanger sequencing of cDNA.
