# Supplementary material for: The Expression of a Germline Fusion Gene Involving a Protein-Coding and a Long Non-Coding RNA Gene Results in Severe Brain Malformations
Source: Genes (Basel). 2025 May 18;16(5):598. doi: 10.3390/genes16050598 (PMC12111516; doi:10.3390/genes16050598)
Supplement: Supplementary file 1 [file genes-16-00598-s001.zip › genes-3644737-supplementary.pdf]

Supplementary Table S1. Primer sets for qRT-PCR from DNA

Primer labelling	Sequence (5'-3')	Length	Tm
CPMER_I2_f (outside the deleted region)	CGCCTCATAAACCCTCTGC	19 nt	60.7 °C
CPMER_I2_r (outside the deleted region)	CCTGTCCTGAGTCCATGAGG	20 nt	60.7 °C
CPMER_E1_f (within the deleted region)	TGGTTGAATGAATGTACCTTAGGAG	25 nt	60.6 °C
CPMER_E1_r (within the deleted region)	CATGCTGGGATTGATTTGTG	20 nt	59.9 °C
Intergenic_f (within the deleted region)	CAGTCTTCGGGGAGAGGAG	19 nt	59.9 °C
Intergenic_r (within the deleted region)	TCTGCTTCTTCTCTGCATGG	20 nt	59.3 °C
MN1_E2_f (within the deleted region)	CCCATTATGGTGTCCCTCAG	20 nt	60.2 °C
MN1_E2_r (within the deleted region)	GATGCTGAGGCCTTGTTTG	19 nt	59.4 °C
MN1_I1_f (within the deleted region)	CACACAGCAAGCAAGGTGAC	20 nt	60.5 °C
MN1_I1_r (within the deleted region)	CCACCTGCTTCCTAGCACAC	20 nt	60.9 °C
MN1_E1_f (outside the deleted region)	GCAGCATGGTGTGTTCTTTG	20 nt	60.3 °C
MN1_E1_r (outside the deleted region)	CTGGGGAGGCTGCATTAAC	19 nt	60.6 °C
PITPNB_E9_f (outside the deleted region/control)	GTGGTGGGGACTGCAAAG	18 nt	60.1 °C
PITPNB_E9_r (outside the deleted region/control)	AATAGCTAGCTCCGATCTCATCC	23 nt	60.2 °C

f = forward; r = reverse; nt = nucleotides; Tm = melting temperature

Supplementary Table S2. Primer sets for qRT-PCR in quantitative RNA analysis

Primer labelling	Sequence (5'-3')	Length	Tm
rt_MN1_E1_f	CCAACGTCTTGTCGTCGTC	19 nt	60.3 °C
rt_MN1_E1_r	AGCACCATTGACCTGGACTC	20 nt	60.1 °C
rt_MN1_E2_f	CACAGACGACGTGGGTGAC	19 nt	61.3 °C
rt_MN1_E2_r	AGCCACGAATGTCCCAAATC	20 nt	62.2 °C
rt_MN1_E1_v2_f	GACGACGACAAGACGTTGG	19 nt	60.3 °C
rt_MN1_E2_v2_r	GATGCTGAGGCCTTGTTTG	19 nt	59.4 °C
rt_MN1_E1_v3_f [fusion transcript 1]	CTGGGGTCAGGTCTTCAGTG	20 nt	60.7 °C
rt_CPMER_E2_r [fusion transcript 1]	GACGACGACAAGACGTTGG	19 nt	60.3 °C

rt = RT-PCR primer; f = forward; r = reverse; nt = nucleotides; Tm = melting temperature

Supplementary Table S3. Primer sets for Sanger sequencing of cDNA

Primer labelling	Sequence (5'-3')	Length	Tm
sp_MN1_E1_v2_f [positive control]	AGGCAGCAGTTCAGCATCTC	20 nt	60.7 °C
sp_MN1_E1_r [positive control]	CCAACGTCTTGTCGTCGTC	19 nt	60.3 °C
sp_MN1_E1_f	GTGGCAAGAAGGGTGAGTG	19 nt	59.2 °C
sp_CPMER_E2_r	ACGCTGCCAAAGGTCTTTTC	20 nt	61.7 °C
sp_MN1_E1_f	GTGGCAAGAAGGGTGAGTG	19 nt	59.2 °C
sp_CPMER_E5_r	AGCTAGGGCTGGCTGGTC	18 nt	60.5 °C
sp_CPMER_E2_f	CCCCAGCGCTTAATTATTTC	20 nt	58.7 °C
sp_CPMER_E3_r	GACATTCTGGGTCCATCTCC	20 nt	59.3 °C
sp_CPMER_E3_f	GAACTGGCCAAATGTGAAGG	20 nt	60.5 °C
sp_CPMER_E4_r	TCACAGTGGTGTTGGTCTCAC	21 nt	59.6 °C
sp_CPMER_E2_f	CCCCAGCGCTTAATTATTTC	20 nt	58.7 °C
sp_CPMER_E4_r	TCACAGTGGTGTTGGTCTCAC	21 nt	59.6 °C

sp = sequencing primer; f = forward; r = reverse; nt = nucleotides; Tm = melting temperature
